# How large is the periablational zone after radiofrequency and microwave ablation? Computer-based comparative study of two currently used clinical devices

**DOI:** 10.1080/02656736.2020.1823022

**Published:** 2020

**Authors:** Macarena Trujillo, Punit Prakash, Pegah Faridi, Aleksandar Radosevic, Sergio Curto, Fernando Burdio, Enrique Berjano

**Affiliations:** a BioMIT, Department of Applied Mathematics, Universitat Politècnica de València, Valencia, Spain;; b Mike Wiegers Department of Electrical and Computer Engineering, Kansas State University, Manhattan, KS, USA;; c Radiology Department, Hospital del Mar, Barcelona, Spain;; d Department of Radiation Oncology, Erasmus MC Cancer Institute, Rotterdam, The Netherlands;; e Department of Surgery, Hospital del Mar, Barcelona, Spain;; f BioMIT, Department of Electronic Engineering, Universitat Politècnica de València, Valencia, Spain

**Keywords:** Microwave ablation, moderate hyperthermic heating, periablational zone, radiofrequency ablation, thermal ablation, tumor ablation

## Abstract

**Purpose:**

To compare the size of the coagulation (CZ) and periablational (PZ) zones created with two commercially available devices in clinical use for radiofrequency (RFA) and microwave ablation (MWA), respectively.

**Methods:**

Computer models were used to simulate RFA with a 3-cm Cool-tip applicator and MWA with an Amica-Gen applicator. The Arrhenius model was used to compute the damage index (Ω). CZ was considered when Ω> 4.6 (>99% of damaged cells). Regions with 0.6<Ω< 2.1 were considered as the PZ (tissue that has undergone moderate sub-ablative hyperthermia). The ratio of PZ volume to CZ volume (PZ/CZ) was regarded as a measure of performance, since a low value implies achieving a large CZ while keeping the PZ small.

**Results:**

Ten-min RFA (51 W) created smaller periablational zones than 10-min MWA (11.3 cm^3^ vs. 17.2 22.9 cm^3^, for 60 100 W MWA, respectively). Prolonging duration from 5 to 10 min increased the PZ in MWA more than in RFA (2.7 cm^3^ for RFA vs. 8.3–11.9 cm^3^ for 60–100 W MWA, respectively). PZ/CZ for RFA were relatively high (65–69%), regardless of ablation time, while those for MWA were highly dependent on the duration (increase of up to 25% between 5 and 10 min) and on the applied power (smaller values as power was raised, 102% for 60 W vs. 81% for 100 W, both for 10 min). The lowest PZ/CZ across all settings was 56%, obtained with 100 W-5 min MWA.

**Conclusions:**

Although RFA creates smaller periablational zones than MWA, 100 W-5 min MWA provides the lowest PZ/CZ.

## 1. Introduction

**Figure 1.**
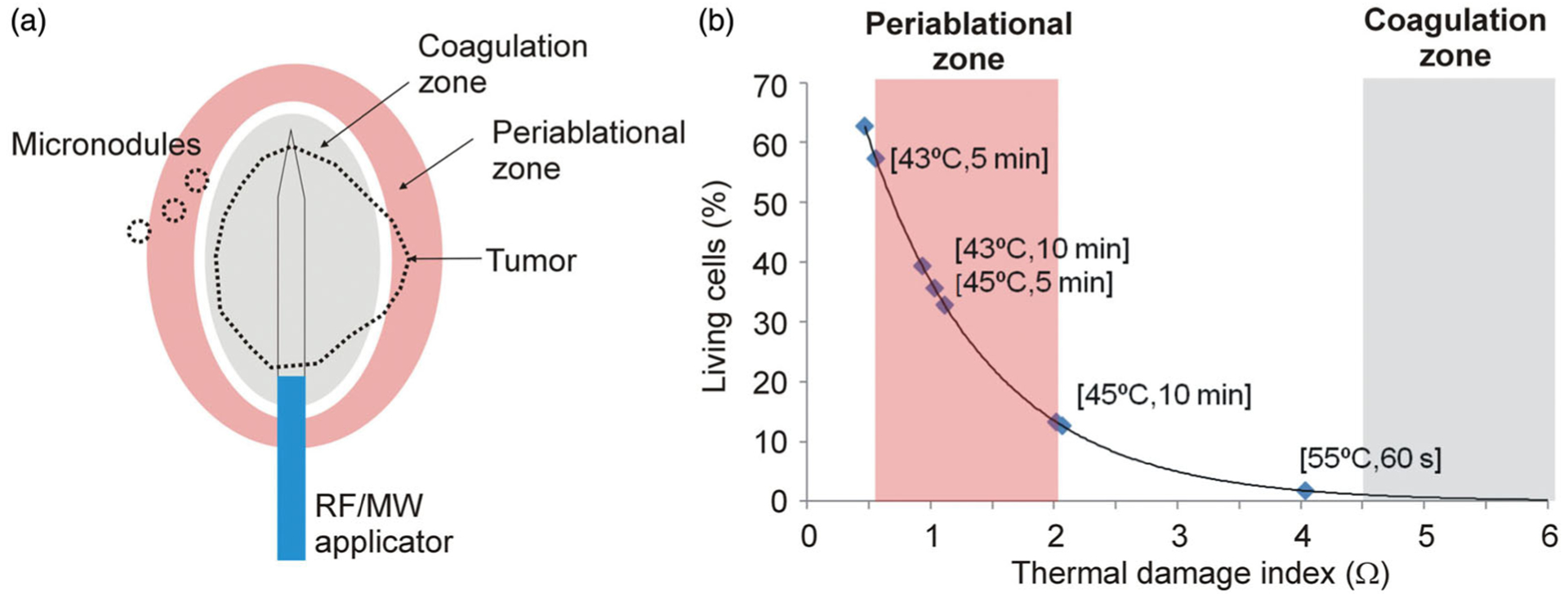
(a) Moderate hyperthermic heating occurs in periablational zone and has been related with tumor cell activity [1]. This heating could affect either a tumor area outside the thermal coagulation zone (which is completely destroyed) or nearby micronodules. (b) Relation between percentage of living cells after heating and index Ω obtained from the Arrhenius damage model, which associates temperature with exposure time using a first-order kinetics relationship. Periablational zone was assumed to be between Ω=0.6 and Ω=2.1 (values derived from experimental data in [1], see text for details), while coagulation zone was defined by the Ω=4.6 contour, which represents 99% probability of cell death.

Energy-based high-temperature ablative therapies such as radiofrequency ablation (RFA) or microwave ablation (MWA) have demonstrated their ability to thermally destroy tumors by creating a coagulation zone that covers a 0.5–1 cm safety margin around the entire tumor. However, evidence is now emerging that any tumor regions that do not reach ablative temperatures may be subsequent promoters of tumor growth [1]. This area, called the *periablational zone*, is always present around the coagulation zone (Figure 1(a)). Even when a previously identified tumor is completely destroyed, the presence of (previously unidentified) satellite micronodules in its vicinity involves a risk when only subjected to moderate heating, i.e., when the micronodules are inside the periablational zone (Figure 1(a)). The ideal ablative technique therefore should be able to create a coagulation zone around the entire tumor plus a 0.5–1 cm margin while keeping the periablational zone beyond the margin as small as possible. The ratio of periablational zone volume to coagulation zone volume could be considered as a measure of value, since an ideal ablative technique should be able to create a large coagulation zone while keeping the periablational zone as small as possible, i.e., the best technique is the one with the lowest PZ/CZ ratio.

Several published studies have compared the outcomes following RFA and MWA. Bench tests [2–5], pre-clinical [6,7] and clinical studies [6] have compared coagulation zone size and treatment outcomes after both energy modalities. While some recent pre-clinical and clinical studies have compared the pro-tumorigenic effects of RFA and MWA [8–10], only one recent *ex vivo* study indirectly compared the volume of the RFA/MWA transition zone, its relationship with residual thermal energy after ablation, and the effect of subsequent cooling on reducing the transition zone [11].

There remains a gap in our knowledge of the size of the periablational zone after thermal ablation in general and the comparative extent of the periablational zone after RFA and MWA. Computer models were thus used to study the thermal performance of two commercial devices widely used for radiofrequency (RFA) and microwave ablation (MWA), respectively, and to compare the size of the periablational zones created by each device.

## 2. Methods

### 2.1. Modeling of periablational and coagulation zones

Computer modeling is widely used to evaluate specific issues of energy-based ablative techniques. Both the physical equations on which they are based and the mathematical framework used to obtain the solutions are now well established and many of them have been validated in terms of coagulation zone size. This size can be estimated by the Arrhenius damage model, which associates temperature with exposure time by a first-order kinetics relationship [12]. This model provides a damage index Ω which is related to the percentage of living cells after the tissue has undergone heating. As Ω>4.6 means that more than 99% of the cells have been irreversibly destroyed (see Figure 1(b)), the Ω=4.6 contour is often used to compute the size of the coagulation zone.

As yet, no thresholds have been established for assessing the extent of the periablational zone based on Ω. However, although there is still no accurate assessment of the scope of this entire area, there do exist experimental data that provide some insight on the subject and may be used as a guide in selecting suitable thresholds; for example, the study by Markezana et al. [1] reports a statistically significant accelerated growth of tumor cells subjected to moderate hyperthermia, especially when heated to 43 and 45 °C for 5 and 10 min. We used these four combinations of temperature–time values to estimate the range of Ω to define the periablational zone by means of the Arrhenius damage model with the following parameters: frequency factor *A* = 7.39×10^39^ s^−1^ and activation energy Δ*E *= 2.577×10^5^ J/mol [13]. We determined that the periablational zone could be limited to between Ω=0.6 and Ω=2.1 (see Figure 1(b)). As 55% of cells are still viable at a damage index of Ω=0.6 and 12% at Ω=2.1, this means that the periablational zone includes both viable cells and those damaged by heating, which can range from around 55% at Ω=0.6 to 88% at Ω=2.1. It must be recognized that the entire periablational zone is composed of cells subjected to moderate hyperthermic heating and is not necessarily limited to the thermal doses considered by Markezana et al. [1], and that if different Ω values were to be found in future studies this would mean redefining the periablational zone and changing some of the conclusions.

### 2.2. Modeling MWA and RFA applicators

The RFA and MWA models were based on those described in [14] and [15]. The geometry of both models was comprised of an applicator (RF electrode or MW antenna) surrounded by a cylinder of liver tissue. For RFA we modeled a Cool-tip applicator (Covidien, Boulder, CO, USA), which is a conventionally cooled, needlelike, 1.5 mm diameter 17 G electrode with a 3 cm active tip, as described in [14]. For MWA we modeled a 20 mm long, 14 G applicator equipped with a mini-choke which mimicked the HS Amica-Gen device (HS AMICA PROBE, HS Hospital Service, Aprilia, Italy) described in [15]. As the geometries presented axial symmetry a two-dimensional analysis was possible.

Both models solved a thermal problem coupled with an electrical (RFA) or electromagnetic (MWA) problem. To solve the thermal problem we used the Bioheat equation modified by the enthalpy method to take vaporization into account and ignored the metabolic heat, which is negligible in both RFA and MWA. The governing equation for the thermal problem was therefore:

(1) $$\frac{\partial \left(\rho h\right)}{\partial t}=\nabla \cdot \left(k\nabla T\right)+q+Q_{p}$$

where *ρ* (kg/m^3^) is tissue density, *h* (J/kg·K) enthalpy, *k* (W/m·K) thermal conductivity, *T* (° C) temperature, *t* (s) time, *q* the heat source and *Q*_*p*_ heat loss by blood perfusion. For biological tissues enthalpy is related to tissue temperature by the following expression [16]:

(2) $$\frac{\partial \left(\rho h\right)}{\partial t}=\frac{\partial T}{\partial t}\cdot \{\begin{array}{ll} \rho_{l}c_{l} & 0<T\leq 99{}^{\circ}C\\ h_{fg}C & 99<T\leq 100{}^{\circ}C\\ \rho_{g}c_{g} & T>100{}^{\circ}C \end{array}$$

where *ρ*_*i*_ and *c*_*i*_ are density and specific heat of tissue respectively at temperatures below 100 °C (*i=l*) and at temperatures above 100 °C (*i* = *g*), *h*_*fg*_ is the product of water latent heat of vaporization and water density at 100 °C, and *C* is tissue water content inside the liver (68%) [17].

In both the RFA and MWA models *q* represented the time average power absorption. In the MWA model *q* was computed from the distribution of the electrical field vector $\vec{E}\left(\text{V}/\text{m}\right)$ as follows:

(3) $$q=\frac{1}{2}\sigma_{e}{\left|\vec{E}\right|}^{2}$$

where *σ*_*e*_ (S/m) is the (effective) conductivity at 2.45 GHz and $\left|\vec{E}\right|$ is the Euclidean norm of $\vec{E}$ (where the *x*, *y*, and *z* components of this vector are peak values). The distribution of $\vec{E}$ was calculated by solving Maxwell’s equations. In contrast, quasi-static approximation was used in the RFA model, which involved replacing all the RF electrical variables (including $\vec{E}$) by DC variables with the same value as the root-mean-square value of the RF signals. *q* was therefore computed as follows:

(4) $$q=\sigma {\left|\vec{E}\right|}^{2}$$

where *σ* is the conductivity at 500 kHz and $\left|\vec{E}\right|$ is the Euclidean norm of $\vec{E}$ (where the x, y, and z components of this vector are equivalent to the root-mean-square value of the RF signal). The electrical field vector was obtained from $\vec{E}=-\nabla V$, *V* being the voltage, which was obtained from the governing equation ∇·(*σ*(*T*)∇*V*)=0.

The blood perfusion term *Q*_*P*_ was obtained from

(5) $$Q_{p}=\beta \rho_{b}c_{b}\omega_{b}\left(T_{b}-T\right)$$

where ω_*b*_ is the blood perfusion coefficient (0.019 s^−1^), ρ_*b*_ and *c*_*b*_ are the blood density and specific heat, respectively, *T*_*b*_ is the temperature of the arterial blood (37 °C) and *β* is a coefficient that modifies blood perfusion with tissue damage: *β* = 0 for Ω≥4.6, and *β*=1 for Ω<4.6.

Both models (RFA and MWA) included the same tissue type with the characteristics described in [14]. Table 1 summarizes the characteristics of all the materials in the model [14,15,18–20]. The changes in the tissue electrical properties were modeled using the equations proposed in [14] for RFA:

(6) $$\sigma \left(T\right)=\{\begin{array}{l} 0.19\;{\text{e}}^{0.015\left(T-37\right)}\;0\leq \text{T}<99{}^{\circ}\text{C}\\ 0.19\cdot 2.5345\;99\leq \text{T}\leq 100{}^{\circ}\text{C}\\ 0.19\cdot 2.5343-0.50183\left(T-100\right)\;100<\text{T}<105{}^{\circ}\text{C}\\ 0.19\cdot 2.5345\times 10^{-2}\text{ T}>105{}^{\circ}\text{C} \end{array}$$

and in [15] for MWA:

(7) $$\varepsilon_{r}\left(T\right)=44.3\left(1-\frac{1}{1+{\text{e}}^{5.223-0.524T}}\right)$$

(8) $$\sigma_{e}\left(T\right)=1.8\left(1-\frac{1}{1+{\text{e}}^{6.583-0.598T}}\right)$$

Null flux was set as a boundary condition in the symmetry axis for the thermal, electrical and electromagnetic problems. A constant temperature of 37 °C (same as the initial) was set at the rest of the boundaries for the thermal problem. The RF electrode’s cooling effect was modeled by Newton’s law of cooling using a thermal convection coefficient of 3127 W/K·m^2^ [14] and a coolant temperature of 5 °C. The condition of 0 V was set at the top and bottom boundaries for the RFA electrical problem to mimic the dispersive electrode and an electrical insulation condition was set in the remaining boundary. A first-order electromagnetic scattering boundary condition was applied in the MWA electromagnetic problem at the outer boundaries together with an initial electric field value of 0 V/m.

The MWA input power was specified as a coaxial port boundary condition at the top of the antenna and in RFA a constant voltage was set at the electrode boundaries. RFA was modeled with a typical clinical protocol based on 90 V pulses, while MWA was modeled with a constant power protocol of 60, 80 and 100 W values, which are typically used in clinical practice [21,22]. Note that these values are the power at the applicator input and may not coincide with those reported in clinical studies in which the reported power may be the MWA generator output power. We modeled two ablation durations: 5 and 10 min. The models were built and simulated with Comsol Multiphysics software (COMSOL, Burlington, MA, USA) using a 2 D axial symmetry geometry. The coagulation and periablational zone volumes were directly calculated by this software by integrating the 2 D zones obtained in each simulation across the azimuth angle. The volumes were computed at two different time points: immediately after terminating ablation (i.e., at 5 and 10 min after the onset of ablation) and 10 min after ablation ended (i.e., 15 and 20 min after onset of ablation). The additional growth of the coagulation zone after power is switched off is due to thermal latency and is especially relevant at very short ablation times [23]. Although we have previously demonstrated that thermal latency is not significant in the case of 4 min RFA (less than 5% growth in diameter) [24], the impact of thermal latency after MWA on the ablation and periablational zones has not yet been reported in the literature.

Since the ideal ablative technique should be able to create a coagulation zone over the entire tumor plus a margin of healthy tissue while keeping the periablational zone as small as possible, we compared the ratio of the volumes of both zones to assess their respective merits.

## 3. Results

**Figure 2.**
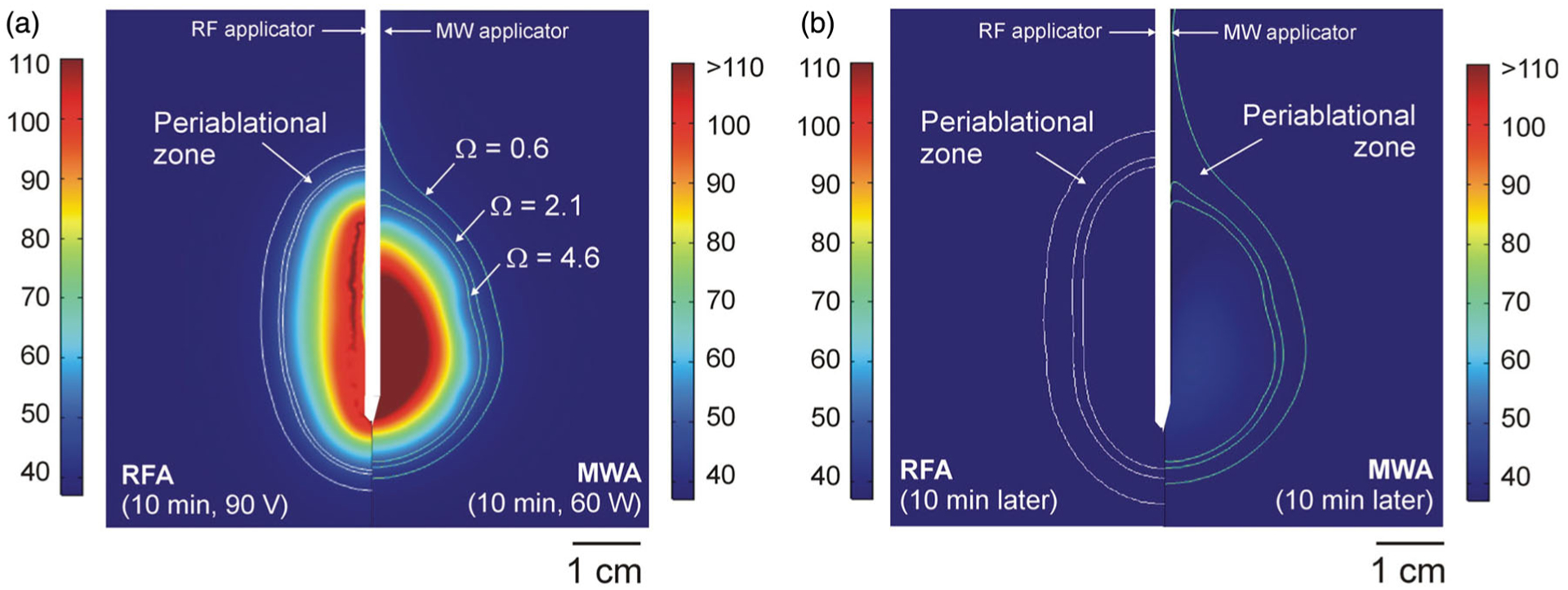
Temperature distributions computed from RFA(Cool-tip applicator, pulsed protocol, 10 min) and MWA (Amica-Gen applicator, 60 W continuous application, 10 min) just after switch-off (a) and 10 min later (b). White lines represent limits of coagulation zone (Ω>4.6) and periablational zones (0.6 < Ω < 2.1). (Scale in °C; MWA temperatures exceeded 110 °C).

Figure 2 shows the temperature distributions obtained with RFA vs. MWA (60 W) after a 10 min ablation and 10 min after switch-off. A similar power level was applied in both cases (mean RFA power 51 W). While the maximum temperature during RFA was 106 C, this was higher in MWA (142 °C at 60 W and 152 °C at 100 W). Both cases presented the typical coagulation morphology: ellipsoidal in the case of RFA and a little more spherical in MWA, except for the slight extension along the applicator shaft toward the connector. The RFA periablational zone was also ellipsoidal around almost the entire coagulation zone. The MWA periablational zone surrounded the coagulation zone, although the axial extension along the applicator shaft was more pronounced than in the coagulation zone. Figure 2(b) shows the temperature distributions 10 min after switch-off. In both cases tissue temperature had almost returned to the initial value.

**Figure 3.**
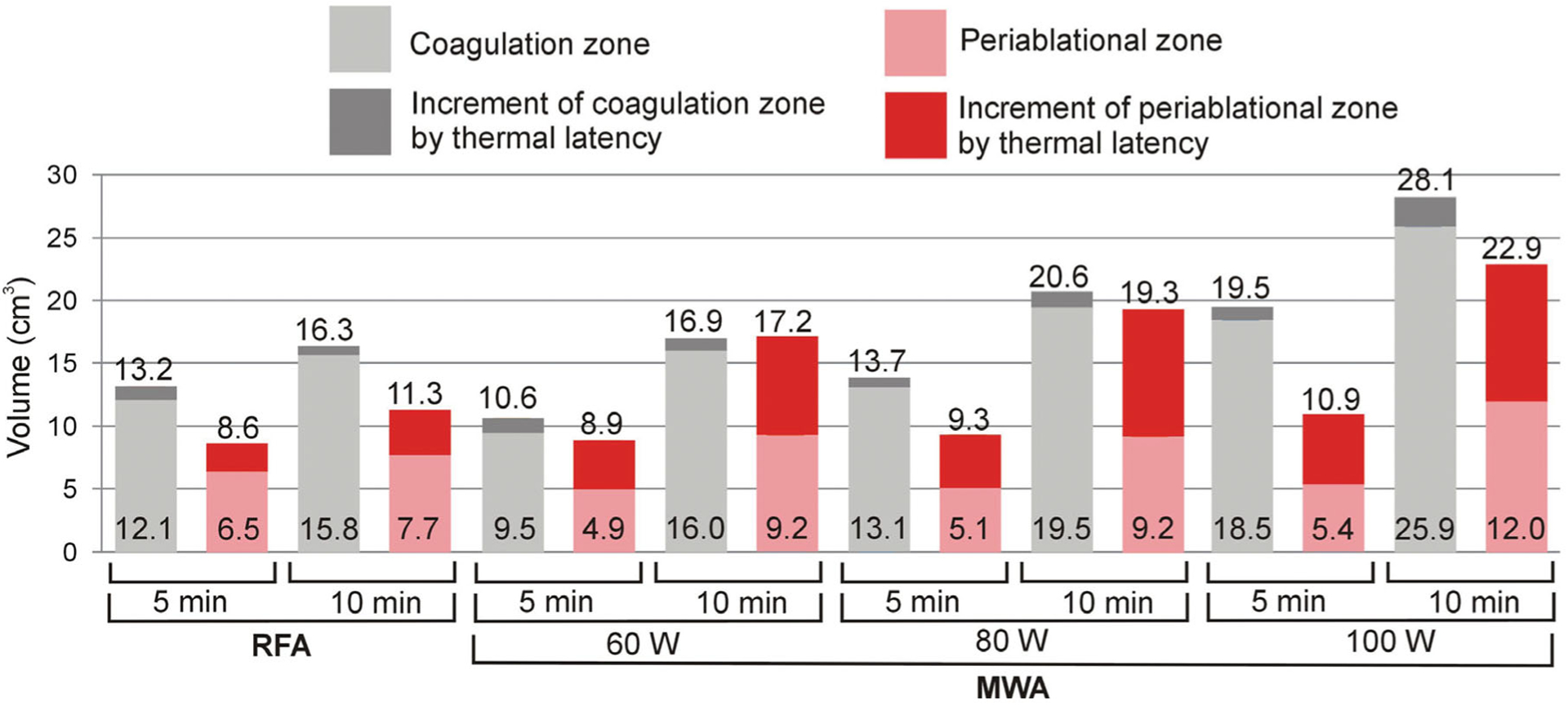
Coagulation and periablational zone volumes in RFA/MWA simulations. Lighter color bars and bottom values are the volumes computed just after switch-off. Darker color bars represent the increase in volume 10 min after switch-off. Upper values give total volumes after this 10-min period.

Figure 3 shows coagulation and periablational volumes of all the cases considered. The following coagulation characteristics were noted: (1) prolonging ablation time from 5 to 10 min results in a greater increase in the MWA coagulation zone (increment of 3.1 cm^3^ for RFA vs. 6.3, 6.9 and 8.6 cm^3^ for 60, 80 and 100 W MWA, respectively); and (2) both techniques create similar coagulation volumes at similar power levels (16.9 cm^3^ at 60 W constant power vs. 16.3 cm^3^ at 51 W mean power, both after 10 min ablation), while MWA provides a larger transverse diameter than RFA (3.20 cm vs. 2.56 cm, both after 10 min ablation).

The periablational zones in Figure 3 show that: (1) RFA creates smaller periablational zones than MWA after 10 min ablation and also 10 min later (11.3 cm^3^ vs. 17.2, 19.3 and 22.9 cm^3^, for 60, 80 and 100 W MWA, respectively); and (2) as with the coagulation zone, prolonging ablation time from 5 to 10 min creates a larger periablational zone in the case of MWA (increment of 2.7 cm^3^ for RFA vs. 8.3, 11.9 and 12.0 cm^3^ for 60, 80 and 100 W MWA, respectively).

**Figure 4.**
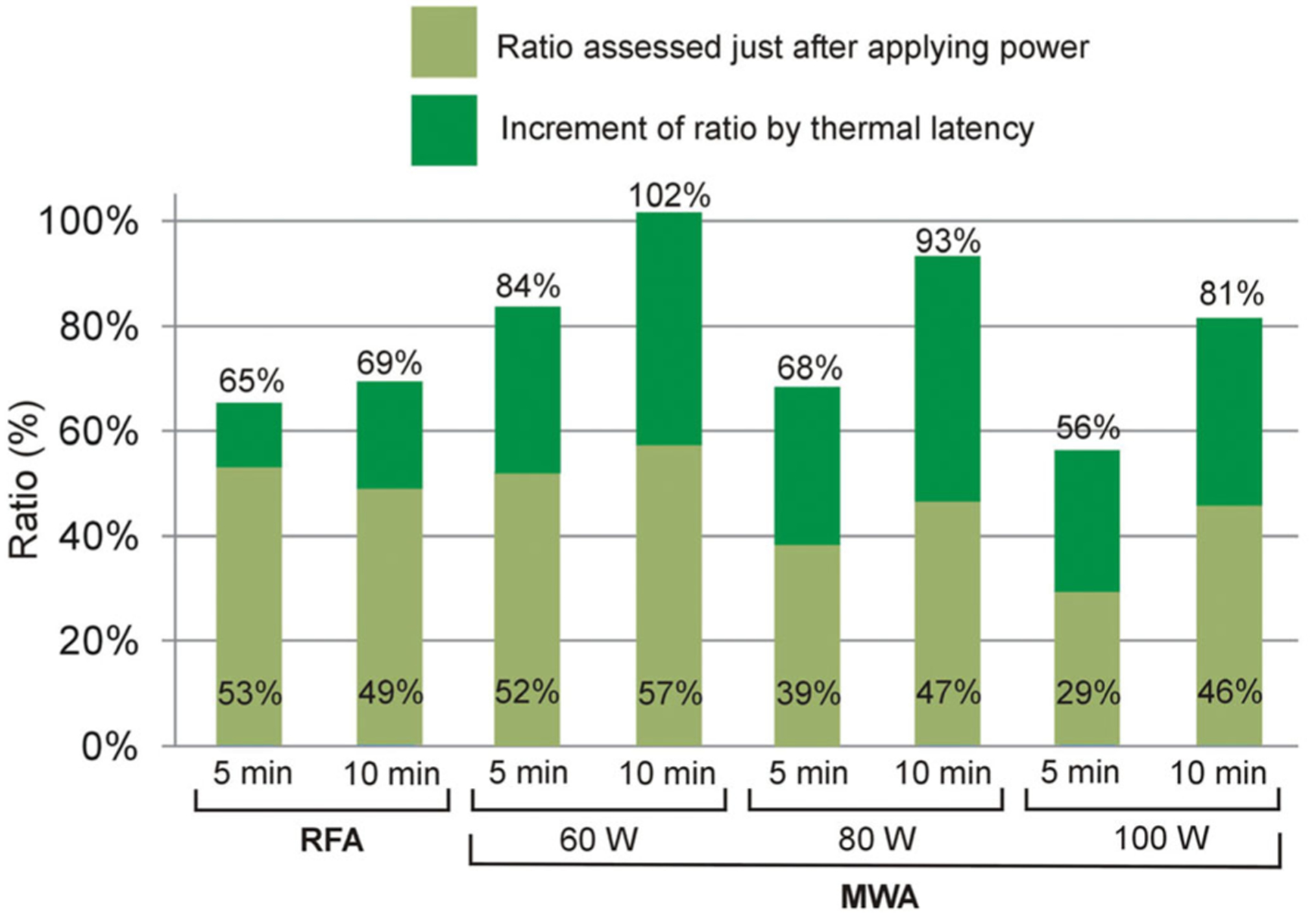
Periablational/coagulation volume ratios in RFA/MWA simulations. Lighter color bars and bottom values are the ratios computed just after switch-off. Darker color bars represent the increased ratio 10 min after switch-off. Upper values show the ratio after this 10-min period.

Figure 4 shows the periablational/coagulation volume ratios for each energy setting just after switch-off and 10 min later. The findings can be summarized as follows: (1) the RFA ratios are relatively high (65–69%) regardless of ablation time (only 4% increase between 5 and 10 min); (2) while the MWA ratio is highly dependent on the duration, with an increase of 18, 25 and 25% between 5 and 10 min at 60, 80 and 100 W, respectively; (3) the MWA ratios are also highly dependent on applied power, with values getting smaller as power is raised (e.g., 102% for 60 W vs. 81% for 100 W, both for 10 min); and 4) the lowest ratio was obtained at high-power short-duration MWA (100 W, 5 min).

## 4. Discussion

As no experimental studies have been published to date that quantify the volume of the periablational zone created by RFA and MWA procedures, we cannot compare our computer modeling results with experimental data. However, they can be compared with other commonly reported experimental parameters such as coagulation diameter and maximum temperature at points near the applicator. Our results show that maximum temperatures during MWA (152 °C for 100 W and 10 min) were significantly higher than those reached in RFA (106 °C for 10 min). There are few existing reports on tissue temperatures during *in vivo* MWA. Laeseke et al. [2] measured temperatures of ~125 °C at 10 mm from the antenna after 9 min of 90 W MWA in *in vivo* porcine kidney. Brace et al. [25] measured temperatures closer to the antenna (5 mm) and found values >150 °C after 9 min of 60 W MWA in *in vivo* porcine kidney. The same studies reported RFA temperatures limited to <100 °C [2,25]. Curto et al. [26] measured temperatures of up to 120 °C at 5 mm from the antenna at a modest power level (30 W) in *ex vivo* porcine muscle. Since the temperature gradients next to the antenna are quite steep, temperatures in the range of 150 °C (or even higher) are plausible closer to the antenna.

Most experimental studies simply report coagulation zone axial and transverse diameters. Some studies calculate the volume from the values of the diameters, while very few use the volumetric techniques used in the present study. This was why our results were compared with previous *in vivo* studies in terms of transverse diameters. Table 2 compares the RFA and MWA coagulation transverse diameters computed after 10-min latency with those reported in experimental studies under similar conditions (*in vivo*) [27–38] (in the case of RFA experimental results of 12-min instead of 10 min ablations, since this is much more common in clinical practice). Despite the dispersion of the experimental results, computer models in general are capable of providing values within the ranges cited in the literature. As computational models therefore come quite close to reproducing maximum temperatures and coagulation volumes, it seems reasonable to assume that the same can be said of the periablational zones.

Our results show that extending ablation time from 5 to 10 min can enlarge the coagulation zone during MWA but not during RFA, a phenomenon that we have previously seen in RFA simulations [14]. This is possibly due to the high impedance of the desiccated tissue after roll-off. However, as the electromagnetic power absorption in tissue during MWA is not determined by the flow of electric current, power continues to be deposited even when the tissue near the antenna has already become desiccated.

**Figure 5.**
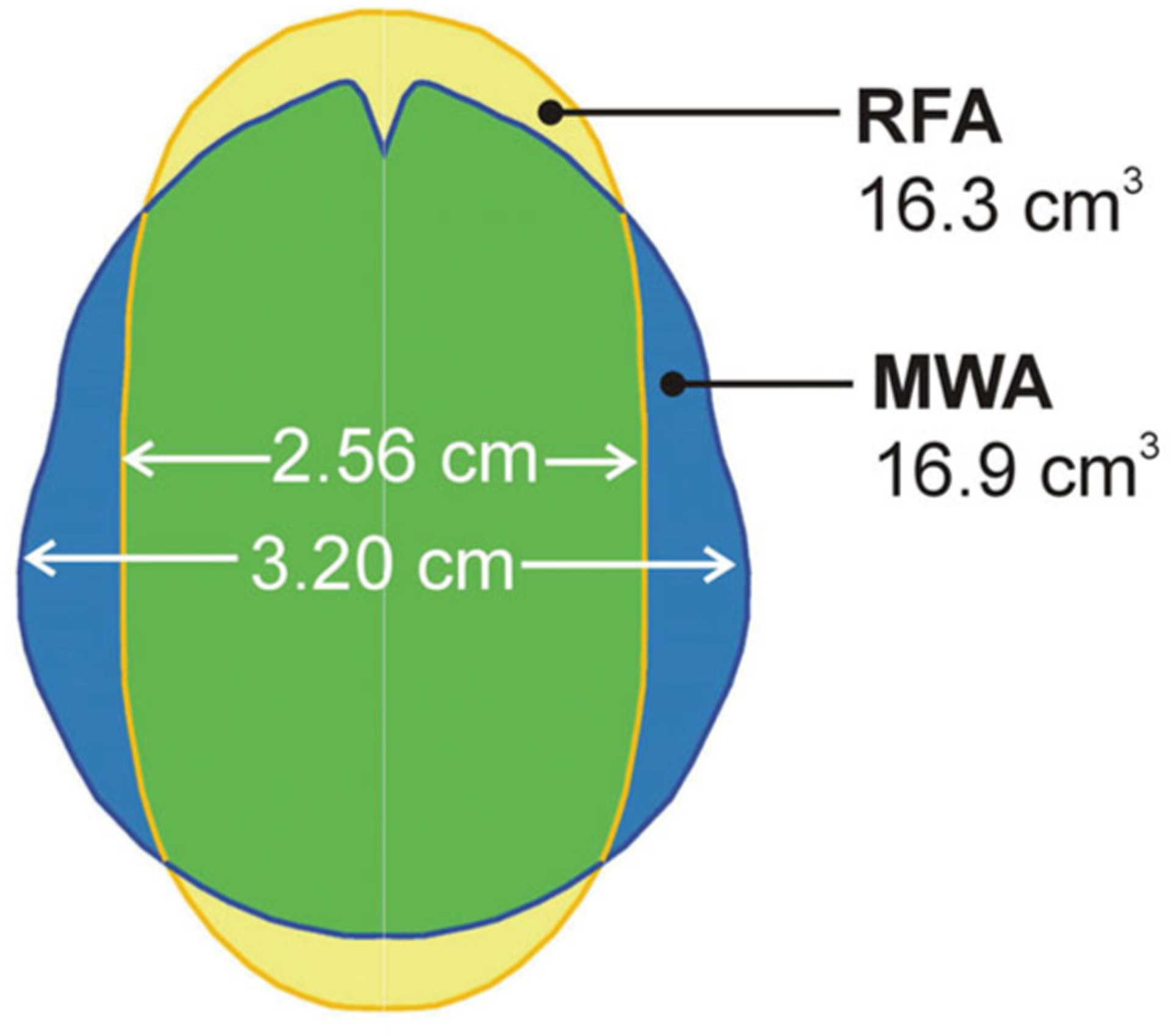
Overlapping coagulation zones after 10 min of RFA (~51 W) and MWA (60 W). Note that while the volumes offer similar values, the MWA transverse diameter is larger.

An interesting finding was that MWA and RFA create similar coagulation volumes at similar power levels. As far as we know, only the study by Andreano et al. [39] compared RFA and MWA at the same power level. Although they concluded that MWA creates larger coagulations in an *ex vivo* setup, their study was based only on the measurement of diameters (4.37 vs. 3.39 cm). We also observed these differences of diameters: 3.20 cm vs. 2.56 cm (lower values than in [39] since we modeled an *in vivo* situation). As can be seen in Figure 5, the differently shaped coagulation zones created by both techniques suggest that the MWA transverse diameter is larger but that the volumes are similar. In this regard, the more spherical MWA coagulation zones could be considered an advantage over the more elliptical RFA shapes.

The present results show that the commonly employed RFA protocol (i.e., mean power ~51 W with impedance control) creates smaller periablational zones than MWA in a 10-min ablation procedures (11.3 cm^3^ vs. 17.2–22.9 cm^3^ at 60–100 W). Although this could mean a clear advantage of RFA over MWA, when analyzing the periablational/coagulation volume ratio, high-power MWA (100 W) and short duration (5 min) has the advantage of creating a larger coagulation zone than RFA (19.5 vs. 16.3 cm^3^) and a smaller periablational zone (10.9 vs. 11.3 cm^3^).

Our results show that the ability of 100 W–5 min MWA to rapidly create relatively large coagulation zones seems to be the key to simultaneously achieving large coagulation and small periablational areas. This is in agreement with the study by Cornelis et al. [40], who assessed the transition zone created after MWA, RFA, cryoablation and irreversible electroporation in *in vivo* porcine kidney and liver, and observed that 5 min MWA created narrower transition zones than other techniques. Their ‘transition zone’ is clearly related to the ‘periablational zone’ since both zones are comprised of both viable and necrotic cells. Despite the promising benefits of using high-power and short-duration MWA, other potentially important issues such as the risk of tumor dissemination associated with high local pressure values induced by high-power applications were not considered [41].

Our study was limited to comparing the volume of the coagulation and periablational zones created with two commercial devices, one for radiofrequency (RFA) and one for microwave ablation (MWA). Although distant tumor growth is probably affected by many different factors, the literature suggests that reducing the amount of tumor cells subjected to moderate hyperthermic heating, i.e., making the periablational zones as small as possible, could be beneficial [1]. In this respect and although it is not possible to establish a relationship with our computational results, the study by Velez et al. [10] on a rat tumor model suggests that although both MWA and RFA can increase distant tumor growth (i.e., large periablational areas), higher power and faster heating protocols could potentially mitigate these undesirable effects.

The main limitation of this study is its theoretical character, since it is based on *in silico* models. Although the RFA and MWA models used in this study were similar to other experimentally validated models, and the data presented in Table 2 suggests the validity of the models in terms of predicting coagulation zone (CZ) size, reasonable doubts could arise as to the models’ periablational zone (PZ) prediction accuracy. To address this issue, we analyzed the experimental data from a recent study on MWA that compared simulated transient temperature profiles and ablation zones in *ex vivo* bovine liver tissue vs. 3 D transient temperature profiles and ablation zones measured by MRI thermometry [42]. The analysis of this data (presented as Supplementary material in the Appendix in the [42–44]) suggests a good agreement between the PZ size estimated from computer simulations and experimental measurements. Although the analysis was done exclusively with MWA data, we think that the validity can be extended to RFA since the thermal damage process is governed by the same principles. Computer modeling thus seems to be a suitable tool for studying issues that would be challenging to accurately assess in experiments, such as the relationship between the coagulation zone and the periablational zone at different power levels and times.

Another minor limitation was that we did not consider a defined tumor region, which may have different thermal, electrical, and perfusion characteristics relative to background liver. Although the present results should be considered as preliminary findings, they are the forerunners of future clinical studies on quantifying the periablational and coagulation zones during and after ablation by imaging techniques to obtain thermal maps in real time [45,46].

Finally, only two commercial applicators were simulated, which limits the conclusions to these models only. However, as they are widely used in clinical practice and are considered to yield large ablation zone volumes, they are representative of optimal design in terms of maximizing coagulation zone size. Different results would possibly be obtained with other applicators specifically designed to minimize the periablational zone volume.

## 5. Conclusions

Our study compared the size of the coagulation zone and periablational zone created by two commercial devices, i.e., 3-cm Cool-tip RF applicator vs. 14 G AMICA microwave antenna. Since the periablational zone volume represents the volume of cells subjected to moderate hyperthermic heating it is desirable to maximize coagulation zone volume while minimizing that of the periablational zone. Allowing for the inherent limitations of a computational model, our results suggest that for a 51 W mean power 10 min RFA with a 3-cm Cool-tip RF applicator, coagulation zone volumes could be similar to those obtained by a 60 W 10 min MWA with a 14 G AMICA microwave antenna. Periablational zone volumes could be larger for MWA than for RFA in all cases (5 and 10 min durations, power levels of 60, 80 and 10 W). Highpower short-duration MWA (100 W and 5 min) could provide the lowest periablational/coagulation zone ratio, thus offering advantages over RFA in terms of creating larger coagulation zones while keeping the periablational zone as small as possible.

## Supplementary Material

Supplementary material

## Figures and Tables

**Table 1.** Characteristics of materials used in the computer model [14,15,18–20].

Material	*σ*_*e*_ (S/m)	*ε*_*r*_	*k* (W/m⋅K)	*ρ* (kg/m^3^)	*c* (J/kg⋅K)	*σ* (S/m)
Liver	1.8^(a)^	44.3^(a)^	0.502	1080^(b)^	3455^(b)^	0.19^(a)^
				370^(c)^	2156^(c)^	
Copper	5.87 × 10^7^	1	385	9000	384	
Alumina	0	10	30	3970	875	
PTFE	1.6 × 10^−5^	1.8	0.24	1200	1050	
Stainless steal	1.74 × 10^6^	1	16.2	8000	500	
Plastic			0.026	70	1045	1 × 10^−5^
Electrode			15	8000	480	7.4 × 10^6^

*σ*_*e*_: (effective) conductivity; *ε*_*r*_: relative permittivity; *k*: thermal conductivity; *ρ*: density; *c*: specific heat; *σ*: electrical conductivity.

(a) Measured at 37 °C,

(b) for temperatures between 37 °C and 99 °C,

(c) for temperatures higher than 100 °C.

**Table 2.** Comparison of RFA/MWA coagulation transverse diameters (in cm) computed by FEM (Finite Element Method) and reported in clinical and experimental studies (*in vivo* ablations only) [27–38].

		MWA					
	RFA 10 min (FEM) 12 min (Clin/Exp)	60 W		80 W		100 W	
		5 min	10 min	5 min	10 min	5 min	10 min
FEM	2.56	2.73	3.20	3.06	3.33	3.43	3.78
Clin./Exp.	1.8 [27], 1.85 [28]	2.5–4 [32]	3.1–4.1 [32]	3.12 [36]	2.37 [35]	2.85 [37]	4.9 [32]
	2.0 [29], 2.6 [30,31]	2.2 [27]	3.1 [34]	3.5 [32]	3.8 [32]	3.4 [32]	
			2.35 [35]			3.3 [38]	
