# Supplementary material for: How large is the periablational zone after radiofrequency and microwave ablation? Computer-based comparative study of two currently used clinical devices
Source: Int J Hyperthermia. Author manuscript; Available in PMC 2020 Dec 3. (PMC7714001; doi:10.1080/02656736.2020.1823022)
Supplement: Supplementary material [file NIHMS1648247-supplement-Supplementary_material.pdf]

APPENDIX

Faridi *et al* [42] recently employed simulations using the same model formulation and tissue physical properties as those in the present study to compare simulated transient temperature profiles and ablation zones in *ex vivo* bovine liver tissue vs. 3D transient temperature profiles and ablation zones measured by MRI thermometry and showed that computational models were a valid means of assessing the extent of the coagulation zone (CZ). Experimental assessment of the periablational zone (PZ) requires the spatial measurement of transient temperature profiles, which could be achieved by Faridi *et al's* experimental approach with MRI. We should point out that Faridi's MWA antenna (a custom water-cooled antenna designed for experimental use) was different to the one simulated in the present study (2.45 GHz, AMICA applicator in clinical use). The AMICA applicator and system are not MRI conditional and thus cannot be used within the MRI environment.

Since the computational model formulation and physical tissue property dependencies in Faridi *et al*. are aligned with those used in the present study, we analyzed the MRI thermometry data from the Faridi study to estimate the extent of the PZ (i.e. $0.6 < \Omega < 2.1$) and compared it to the corresponding simulated estimation of PZ size, considering $n = 4$ experiments performed with 30 W at the 2.45 GHz applicator input for 5 min (~60 W at the generator) and another $n = 4$ experiments performed with the same power for 10 min. Figure A1 shows the extent of the simulated $\Omega = 0.6$ and $\Omega = 2.1$ contours for the two power/time combinations. These contours are overlaid on PZ maps as assessed from MRI thermometry.

Briefly, for each experiment, Arrhenius thermal damage calculations were performed on transient temperature profiles measured by MRI thermometry. We compared the simulated PZ vs. the PZ derived from MRI thermometry in each experiment using the Dice Similarity Coefficient (DSC). Table 1A shows the DSC values of the experimental cases compared to the simulations. DSC > 0.7 is generally considered to indicate a good overlap (or good agreement) between two shapes [43,44]. The obtained DSC values were generally greater than 0.7, indicating good matches, except for one outlier at 30 W, 5 min.

The experimentally derived Ω maps were then compared to the PZ threshold (Ω = 0.6 and Ω = 2.1) to create binary images. The binary images from each experimental run (n = 4) and group were then joined to create a composite image in which a pixel value of 4 indicated that the tissue region in the pixel was heated to above the Ω threshold in each of the 4 experiments, while a pixel value of 3 indicated that the tissue region in the pixel was heated to above the Ω threshold in 3 of the 4 experiments, and so on. When comparing the simulated PZ contours with the average of the binarized PZ-thresholded maps (i.e. pixel value = 2) from MRT we again found good agreement (DSC > 0.7) between the simulated and experimentally assessed PZ areas, which suggests that the simulated PZ size agrees reasonably well with the experimentally measured PZ size.

Table A1. Dice Similarity Coefficients (DSC) obtained by comparing experimental (MRI thermometry) and computational PZ results.

**Power and time**	**Ω = 0.6**		**Ω = 2.1**	
	Raw data (n=4)	Average	Raw data (n=4)	Average
30 W, 5 min	0.67, 0.78, 0.79, 0.76	0.80	0.54, 0.70, 0.70, 0.71	0.74
30 W, 10 min	0.77, 0.83, 0.83, 0.85	0.86	0.74, 0.81, 0.80, 0.82	0.83

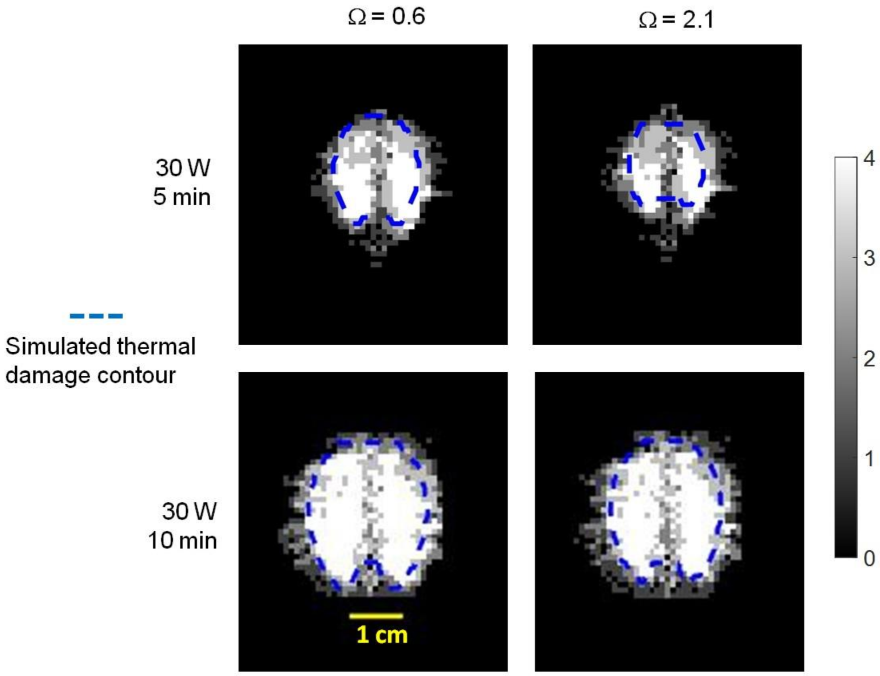

Figure A1 Contours of the limits of the periablational zone (PZ) (i.e. Ω = 0.6 and Ω = 2.1) computed from simulation (dashed blue line) for two MWA power/time settings. These contours are overlaid on PZ maps as assessed by MRI thermometry.
